# Baseline spirometry parameters as predictors of airway hyperreactivity in adults with suspected asthma

**DOI:** 10.1186/s12890-021-01506-6

**Published:** 2021-05-06

**Authors:** Michael Peled, David Ovadya, Jennifer Cohn, Lior Seluk, Teet Pullerits, Michael J. Segel, Amir Onn

**Affiliations:** 1 Institute of Pulmonary Medicine, Chaim Sheba Medical Center, Derech Sheba st. 2, 52621 Ramat Gan, Israel; 2 Sackler Faculty of Medicine, Tel-Aviv University, Tel-Aviv, Israel; 3 Department of Respiratory Care and Rehabilitation, Chaim Sheba Medical Center, Ramat Gan, Israel; 4 Faculty of Medicine, Sahlgrenska Academy, University of Gothenburg, Gothenburg, Sweden; 5 Department of Asthma and Allergology, Sahlgrenska University Hospital, Gothenburg, Sweden

**Keywords:** Asthma, Bronchoconstrictor agents, Methacholine, Spirometry

## Abstract

**Background:**

Methacholine challenge tests (MCTs) are used to diagnose airway hyperresponsiveness (AHR) in patients with suspected asthma where previous diagnostic testing has been inconclusive. The test is time consuming and usually requires referral to specialized centers. Simple methods to predict AHR could help determine which patients should be referred to MCTs, thus avoiding unnecessary testing. Here we investigated the potential use of baseline spirometry variables as surrogate markers for AHR in adults with suspected asthma.

**Methods:**

Baseline spirometry and MCTs performed between 2013 and 2019 in a large tertiary center were retrospectively evaluated. Receiver-operating characteristic curves for the maximal expiratory flow-volume curve indices (angle β, FEV1, FVC, FEV1/FVC, FEF_50%_, FEF_25–75%_) were constructed to assess their overall accuracy in predicting AHR and optimal cutoff values were identified.

**Results:**

A total of 2983 tests were analyzed in adults aged 18–40 years. In total, 14% of all MCTs were positive (PC20 ≤ 16 mg/ml). All baseline spirometry parameters were significantly lower in the positive group (*p* < 0.001). FEF_50%_ showed the best overall accuracy (AUC = 0.688) and proved to be useful as a negative predictor when applying FEF_50%_ ≥ 110% as a cutoff level.

**Conclusions:**

This study highlights the role of FEF_50%_ in predicting AHR in patients with suspected asthma. A value of ≥ 110% for baseline FEF_50%_ could be used to exclude AHR and would lead to a substantial decrease in MCT referrals.

**Supplementary Information:**

The online version contains supplementary material available at 10.1186/s12890-021-01506-6.

## Background

Methacholine challenge tests (MCTs) are used to detect and assess airway hyperreactivity (AHR). MCT has a negative predictive value of almost 90% for the provocative concentration causing a 20% fall (PC20) in forced expiratory volume in one second (FEV1) when PC20 is greater than 16 mg/ml [1], which makes it useful for excluding the diagnosis of asthma, especially in the setting of equivocal spirometry findings, in the presence of typical asthma symptoms. Even though bronchial provocation tests are generally safe, they are time consuming and costly and often require a referral to a specialized testing center. Thus, it is of interest to minimize the amount of testing by predicting which patients will have a negative outcome in the MCT.

Several variables derived from the Maximal Expiratory Flow Volume (MEFV) curve have been suggested as possible predictors of AHR [2]. Two possible candidates are the forced expiratory flow rate between 25 and 75% of vital capacity (FEF_25–75%_) and at 50% of vital capacity (FEF_50%_). They are considered approximate measures of the flow in the peripheral airways and a reduction in either variable may therefore represent airflow limitation in the small airways [3, 4]. A study analyzing AHR in asthmatics showed that FEF_50%_ percent of predicted was the best surrogate marker among all standard lung function variables to predict PC20 < 4 mg/ml [2], including FEV1, forced vital capacity (FVC), FEV1/FVC, and FEF_25–75%_. No studies have investigated the predictive ability of these variables in a general population consisting of both asthmatics and healthy individuals.

In addition to the standard baseline parameters of spirometry, such as FEV1, FVC, FEV1/FVC, FEF_25–75%_ and FEF_50%_, a subjective visual assessment of concave patterns in the curvature of the descending limb of the MEFV may also suggest an obstructive process [5–7]. In 2005, the American Thoracic Society (ATS) and European Respiratory Society (ERS) task force stated that the first sign of airflow obstruction in the small airways on a spirogram is this concavity [8]. Objective measures to assess the curvilinearity of the MEFV curve have been proposed over the decades [6, 9, 10]. In 1988 Kapp et al. defined a new parameter, referred to as angle β, which characterized the shape of the MEFV curve by applying trigonometry to the conventional variables obtained from spirometry. The study further showed that the angle β was significantly lower in individuals with asthma than in healthy individuals [11]. Unfortunately, no reference values have been established for an adult population and the angle β therefore needs to be used with caution.

Since MCTs are expensive and time consuming, it is of importance to identify which individuals are highly unlikely of having a positive test beforehand. By finding an appropriate surrogate marker for AHR, physicians could already identify in a primary care setting which individuals have low probability of asthma and can be spared further testing.

We aimed to investigate the potential use of the angle β and more standard spirometry parameters, such as FEV1 and FVC, as surrogate markers for predicting AHR in a general population, with the objective to elucidate novel ways of excluding AHR and subsequently avoid unnecessary MCTs.

## Methods

### Setting and study participants

This cross-sectional study was based on MCTs performed at the Institute of Pulmonology at Sheba medical center, Israel.

Data from study participants between 18 and 40 years of age who had undergone MCTs between 2013 and 2019 were included in the analysis. Participants were referred to MCT due to symptoms suggestive of asthma where a previous test, such as exercise challenge test or reversibility testing, had been inconclusive, in accordance with the GINA guidelines for asthma diagnosis [12].

### Design

The dataset was randomly split into two cohorts, 75% were assigned to the derivation cohort (n = 2237) and 25% were included in the validation cohort (n = 746). The validation cohort was used to evaluate if the predictive models obtained in the derivation cohort perform similarly for a separate dataset (internal validation).

The following variables were obtained from the dataset and used for analysis: gender, age, weight (kg), height (cm), forced vital capacity (FVC), forced expiratory volume in 1 s (FEV_1_), peak expiratory flow rate (PEFR), forced expiratory flow at 50% of forced vital capacity (FEF_50%_), Forced expiratory flow at 25–75% of forced vital capacity (FEF_25–75%_), the methacholine provocation concentration resulting in a 20% fall in FEV_1_ (PC_20_). Predicted values for FVC, FEV_1,_ Peak expiratory flow rate (PEFR), FEF_25–75%_, and FEF_50%_ were based on reference equations from the European Community of Coal and Steel (ECCS) [13], and were also analyzed according to the reference equations from the Global Lung Function Initiative (GLI) [14]. In addition, the following composite variables were calculated from extracted data: BMI (kg/m^2^), FEV_1_/FVC ratio, FEV1/FVC %predicted, FVC %predicted, PEFR %predicted, FEV1%predicted, FEF_50%_ %predicted and FEF_25–75%_ %predicted.

To quantify the shape of the maximum expiratory flow-volume curve (MEFV), the angle β was calculated using equation in [11]: $\beta=180^{\circ}-{\text{tan}}^{-1}\text{(PEFR-FEF}_{\text{50\%}}/0.5\times\text{FVC)}+ {\text{tan}}^{-1}({\text{FEF}}_{\text{50\%}}/0.5\,\text{FVC)}$. All tan^−1^ values were calculated in degrees and defined as the a ngle formed when projecting a line from the PEFR point to the mid flow point on the X-axis (FEF_50%_) and then connecting that point to the end point of the forced vital capacity (Additional file 1: Fig. S1).

Following ATS guidelines, a negative methacholine challenge result was defined as PC20 > 16 mg/ml, while values of PC20 ≥ 4 to ≤ 16 mg/ml were defined as borderline AHR [15]. For borderline cases the patient’s symptoms were assessed by a physician and asthma treatment was initiated if deemed appropriate. For this reason, PC20 ≤ 16 mg/ml was set as the appropriate cut-off level for this study.

### Statistical analysis

The mean values of continuous variables were compared using two-tailed t-test. Differences in frequencies for categorical variables were analyzed using Chi-squared test. The result for continuous variables was expressed as the mean ± SD and categorical data was presented as number of observations and proportion of observations, in percent. For the derivation cohort, receiver operating curve (ROC) analysis was performed for all lung function parameters individually to determine the usefulness of each parameter for predicting a positive outcome in the MCT. The diagnostic performance of each variable was expressed as area under the curve (AUC). For the parameter with the best predictability, an optimal cut off point was established to optimize sensitivity and specificity. A multivariate analysis was performed using logistic regression; all available parameters were included. Using backward analysis, the number of variables was reduced until the model with the highest discriminative power was retained. ROC curve analysis was also used to verify diagnostic accuracy in the validation cohort. For the whole analysis, a *P* value ≤ 0.05 was considered statistically significant. Microsoft Excel version 16.35 was used to create composite variables and calculation of the angle β. Statistical analyses were performed using Graph Pad Prism version 8.4.0. and RStudio version 1.1.414.

## Results

### Comparison of characteristics in derivation and validation cohort

There was no significant difference in clinical characteristics at baseline between the derivation and validation cohorts (Table 1 according to ECCS equations, Additional file 1: Table S1 according to GLI equations and z-scores; there is no reference for FEF50% in the GLI, thus both FEF50% and the angle β, which is based on FEF50%, are not shown for GLI-based spirometry parameters). The distribution of gender was 81% males with a mean age of 24.2 years in both cohorts, and 14% of both cohorts had a positive outcome (PC20 ≤ 16 mg/ml) in the MCT. The male to female ratio was higher than the ratio in the general population because Sheba Medical Center accepts Israel Defense Forces recruits for asthma assessment. Importantly, gender did not affect the results of all the analyses below, according to subgroup analyses.

**Table 1:** Demographics and pulmonary lung function characteristics at baseline for derivation and validation cohort

	Derivation cohort (n = 2237)		Validation cohort (n = 746)		*P* value
	Mean	SD	Mean	SD	
Age (years)	24.20	3.67	24.20	3.60	0.62
Females	434 (19%)	–	142 (19%)		0.83*
Height (cm)	173.53	8.42	173.87	8.50	0.34
Weight (kg)	69.43	12.45	69.40	12.70	0.96
BMI (kg/m^2^)	22.99	3.44	22.92	3.70	0.67
FEV_1_/FVC (%pred)	83.50	7.50	83.40	7.20	0.61
FEV_1_ (%pred)	94.50	11.30	94.40	11.30	0.92
FEF_50%_ (%pred)	87.80	22.80	87.60	22.30	0.82
FVC (%pred)	99.10	13.00	99.50	13.20	0.49
FEF_25–75%_ (%pred)	86.30	23.10	87.10	23.70	0.43
PC20 ≤ 16 mg/ml (%)	305 (14%)	–	103 (14%)		0.91*
Angle β (°)	186.14	14.80	185.88	14.80	0.68

BMI, Body Mass Index; FEV_1_, forced expiratory volume in 1 s; FVC, forced vital capacity; FEF_50%_, forced expiratory flow at 50% of FVC; FEF_25–75%_, forced expiratory flow at 25–75% of FVC; PC20, Concentration of methacholine causing a 20% decrease in FEV_1_. Relative values of spirometry parameters are given as percentage of the predicted value (% pred)

^*^P value from Chi-squared analysis

### Positive versus negative methacholine challenge test groups: baseline comparisons

In order to determine which clinical parameters might have a predictive value, the derivation cohort was stratified depending on outcome in the MCT and comparisons between the two groups were conducted (Table 2 according to ECCS equations, Additional file 1: Table S2 according to GLI equations and z-scores). The two groups had similar demographical characteristics. In contrast, all baseline spirometry parameters were significantly lower in the group with AHR (PC20 ≤ 16 mg/ml) compared to the group without AHR according to ECCS equations (Table 2). However, FVC% and FEF25–75% were not significantly different between the two groups according to GLI equations (Additional file 1: Table S2).

**Table 2:** Comparison of demographics and baseline spirometry parameters in patients with positive versus negative methacholine challenge test in the derivation cohort

	Methacholine negative (n = 1932)		Methacholine positive (n = 305)		*P* value
	Mean	SD	Mean	SD	
Age (years)	24.10	3.65	24.50	3.80	0.16
Females	367 (19%)	–	67 (22%)	–	0.22*
Height (cm)	173.60	8.29	172.89	9.16	0.18
Weight (kg)	69.53	12.26	68.77	13.50	0.36
BMI (kg/m^2^)	23.01	3.44	22.80	3.46	0.56
Smoking n (%)**					0.26
Non-smoker	176 (67)		42 (67)		
Ex-smoker	12 (5)		6 (9)		
Smoker	75 (28)		15 (24)		
Total	263 (100)		63 (100)		
FEV_1_/FVC ratio (%pred)	84.10	7.40	80.10	7.50	< 0.01
FEV_1_ (%pred)	95.40	11.00	89.10	11.70	< 0.01
FEF_50%_ (%pred)	89.80	22.80	75.10	18.00	< 0.01
FVC (%pred)	99.50	13.00	96.50	13.00	< 0.01
FEF_25–75%_ (%pred)	86.90	23.10	82.70	22.80	< 0.01
angle β (**°**)	186.96	15.01	180.92	12.36	< 0.01

Methacholine positive defined as PC20 ≤ 16 mg/ml; PC20, Concentration of methacholine causing a 20% decrease in FEV_1_. BMI, Body Mass Index; FEV_1_, forced expiratory volume in 1 s; FVC, forced vital capacity; FEF_50%_, forced expiratory flow at 50% of FVC; FEF_25–75%_, forced expiratory flow at 25–75% of FVC; Relative values of spirometry parameters are given as percentage of the predicted value (% pred)

^*^P value from Chi-squared analysis

^**^Smoking status was available for 326 patients in the derivation cohort—263 from the MCT negative patients and 63 from the MCT positive patients

### Predictive value of baseline lung function parameters

**Fig. 1:**
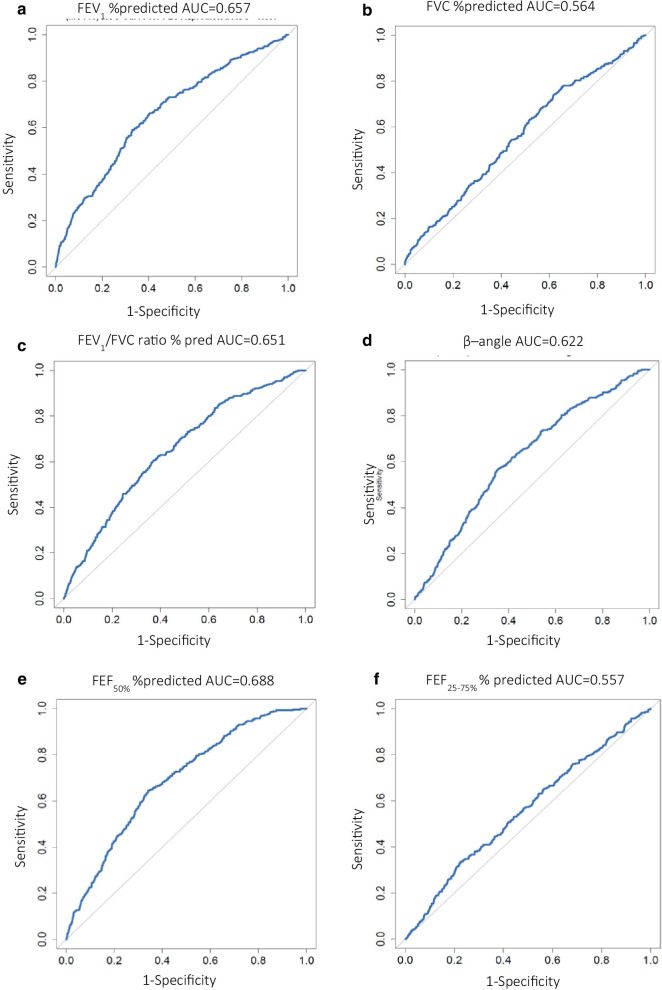
Receiver operating characteristics (ROC) curve for; **a** baseline forced expiratory volume in 1 s**,** FEV1%predicted; **b** forced vital capacity, FVC %predicted; **c** FEV1/FVC; **d** angle β; **e** forced expiratory flow at 50% of FVC, FEF50% %predicted; **f** forced expiratory flow at 25–75% of FVC, FEF25–75% %predicted in the derivation cohort as predictors of methacholine responsiveness. AUC = Area under the curve. Grey line represents the line of unity with AUC = 0.5

Since all baseline lung function parameters were significantly different between the two groups according to ECCS equations, ROC analysis was performed on all spirometry variables in the derivation cohort, to test their predictive value for AHR. The area under the ROC curve (AUC) was calculated in order to assess the usefulness of each parameter for predicting positive outcome (PC20 ≤ 16 mg/ml) in the MCT. Of all the tested parameters, FEF_50%_ %predicted was identified as the best predictor, having the highest diagnostic accuracy of AUC = 0.688 (Fig. 1). Lower predictive values were found for FEV_1_% predicted, FEV_1_/FVC % predicted and the angle β, with AUC of 0.657, 0.651 and 0.622 respectively (Fig. 1a, c, d). Lastly, FVC % predicted and FEF_25–75%_ % predicted were weaker predictors of AHR (Fig. 1b, f). Similar results were found for spirometry parameters that were calculated according to GLI equations (Additional file 1: Fig. S2 and Fig. S3 for z-scores.).

### Threshold values for baseline spirometry parameters

**Fig. 2:**
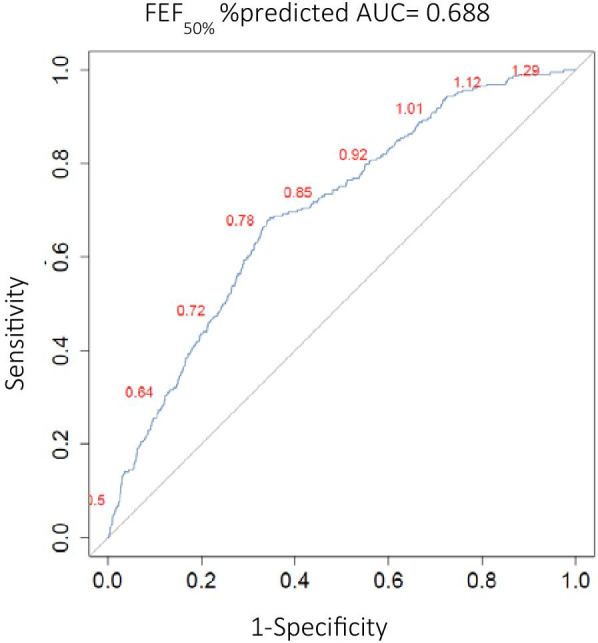
Receiver operating characteristics (ROC) curve for forced expiratory flow at 50% of FVC, FEF_50%_ %predicted, in the derivation cohort as a predictor of methacholine responsiveness. Values in red represent the respective FEF_50%_ %predicted cut-off values. AUC = Area under the curve. Line of unity is represented by a grey line, corresponding to AUC = 0.5

Since this study aims to identify potential baseline parameters that are useful for predicting individuals unlikely of displaying AHR, different cut-off values and their respective specificity and sensitivity were assessed in order to determine what values were clinically useful (Fig. 2). In Table 3, the sensitivity, specificity, positive predictive value (PPV) and negative predictive value (NPV) for different cut-off levels of FEF50% predicted are shown. When employing FEF_50%_ %predicted < 120% as a cut-off level, a sensitivity and specificity of 99.1% and 10% respectively was obtained. When setting the cut-off level for FEF_50%_ %predicted at < 110%, the sensitivity and specificity were 96.6% and 19.3%. To further illustrate the clinical implications of using FEF_50%_ %predicted of < 110% as a threshold value, 395 (17.7%) of all participants in the derivation cohort had a baseline value that was ≥ 110%. Of those only 8 (0.3% of derivation cohort) were false negatives, i.e. displayed AHR (PC20 ≤ 16 mg/ml), and of those only 6 showed significant AHR (PC20 < 4 mg/ml). This means approximately 17.7% of all MCTs can be avoided if FEF_50%_ %predicted of ≥ 110% is used as an exclusion. Since the GLI equations are focused on outcomes recommended by the ATS/ERS guidelines (i.e. FEV1, FVC and FEV1/FVC), and thus do not include FEF_50%_, we have also assessed the ability of FEV1%predicted to predict AHR (Additional file 1: Fig. S4 and Fig. S5 for z-scores, Additional file 1: Table S3). When setting the cut-off level for FEV1%predicted at < 110%, the sensitivity and specificity were 97.9% and 2.8%, respectively, demonstrating reduced specificity compared with FEF_50%_ %predicted. In addition, only 61 (2%) of all participants in the derivation cohort had a baseline FEV1 value that was ≥ 110%. Of those 7 were false negatives, i.e. displayed AHR (PC20 ≤ 16 mg/ml), and of those 5 showed significant AHR (PC20 < 4 mg/ml), again demonstrating that FEV1 has reduced predictive value compared with FEF_50%_.

**Table 3:** Diagnostic performance of baseline FEF_50%_ % predicted for different cut-off values, obtained by ROC analysis to predict methacholine responsiveness (PC20 < 16 mg/ml)

Cut-off (%)	Sensitivity (%)	Specificity (%)	PPV (%)	NPV (%)
FEF_50%_ %predicted				
< 120	99.10	10	11.40	99
< 115	97.80	14.50	11.80	98.30
< 110	96.60	19.30	12.30	97.90

Sensitivity, specificity, positive predictive value (PPV), negative predicted value (NPV)

### Logistic regression model

To create a model that combines several parameters and to detect possible confounding variables, backwards logistic regression was performed with the relevant variables.

**Fig. 3:**
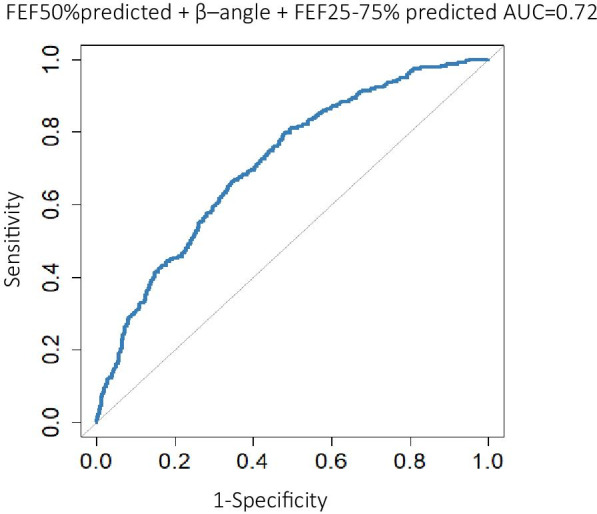
Receiver operating characteristics (ROC) curve for model combining forced expiratory flow at 50% of FVC, FEF_50%_ %predicted, angle β and forced expiratory flow at 25–75% of FVC, FEF_25–75%_ %predicted as a predictor of methacholine responsiveness in the derivation cohort. AUC = Area under the curve. Line of unity is represented by a grey line, corresponding to AUC = 0.5

A predictive model combining FEF_50%_ %predicted with the angle β and FEF_25–75%_ %predicted yielded the highest discriminative power, with an AUC = 0.72 (Fig. 3). The diagnostic accuracy of this model outperforms all single parameter models proposed above. These three baseline parameters were associated with methacholine responsiveness independently of FEV1% predicted, FVC % predicted and FEV1/FVC ratio. The linear combination of the three parameters is depicted in the following equation: the probability for positive

$${\text{MCT16}} = \frac{1}{{1 + e^{{0.69 + 4.3 \times \left( {FEF_{50\%}\,\% predicted} \right) - 0.02 \times Beta\_angle + 0.86 \times \left( {FEF_{25\%-75\%}\,\% predicted} \right)}} }}$$

### Validation cohort

To assess if the previously produced results and predictive models hold true for a separate sample, replication of the analysis on all spirometry variables was performed on the validation cohort. Once again, FEF_50%_ %predicted had the highest AUC of 0.692 (Additional file 1: Fig. S6) and was recognized as the best predictor of AHR. When applying a cut-off value of 110% for FEF_50%_ %predicted in the validation cohort, the sensitivity was 98.1% and the specificity 20.1%, with an accompanying NPV of 98.5. Of all the participants in the validation cohort, 17.6% had an FEF_50%_ %predicted over 110%, meaning those study participants could have been exempt from going through a methacholine provocation. The logistic regression model once again showed a model combing FEF_50%_ %predicted, angle β and FEF_25–75%_ %predicted provided the highest diagnostic accuracy of AUC = 0.73 (Additional file 1: Fig. S7). Overall, the results in the validation cohort were consistent with those from the derivation cohort.

## Discussion

The aim of this study was to investigate baseline spirometry measures as potential predictive markers for absence of AHR in suspected asthmatics. The findings identified FEF_50%_ %predicted as the best parameter to predict absence of AHR, while FVC % and FEF_25–75%_ were poor predictors for a positive MCT. These results are in line with a previous study that found that FEF_50%_ % predicted was the best parameter in order to predict degree of AHR in asthmatics, when PC20 ≤ 4 mg/ml was used as a cutoff for a positive outcome [2]. Another study found that asthmatic subjects with low FEF_50%_ %predicted had significantly higher AHR, independently of FEV1, thus suggesting the contribution of the small airways to the severity of AHR [16]. The main difference and added novelty in our study is the inclusion of non-asthmatics and the potential of using the marker to exclude asthma at an early stage in the diagnostic process. The results indicate the potential of utilizing FEF_50%_ %predicted in a clinical setting to inform physicians if there is any added diagnostic value in referring patients to MCTs. Unfortunately, there are no reference values for FEF_50%_ in the GLI equations, which are now considered as best practice standard, and our results imply that references for this important parameter should be considered in the future for GLI equations. Our analyses also imply that FVC % and FEF_25–75%_ are poor predictors for a positive MCT. Importantly, both FEF_25–75%_ and FEF_50%_ are derived from FVC and thus are sensitive to errors in FVC measurement. Hence, normalization of these flows to FVC may allow improved prediction of AHR.

The discriminatory capability of FEF_50%_ does however have limitations with an overall diagnostic accuracy of 68.8% (AUC = 0.688). This is mainly caused by a large amount of overlap between asthmatics and healthy individuals. This study however emphasizes its value as a negative predictor of AHR and suggests using a threshold value of ≥ 110% of baseline FEF_50%_ %predicted to predict negative outcome in the MCT. When applying this cut-off value to the derivation cohort and validation cohort, almost a fifth of all tests could be avoided which in turn would save valuable time and money, both for health care providers and for individuals.

A model combining FEF_50%_, FEF_25–75%_ and angle β was created by using backward logistic regression analysis on all parameters, and showed a slightly higher predictive ability than just using a single parameter, such as FEF_50%_ %predicted. Since all three parameters have been suggested as markers of small airways obstruction, this model may indicate that obstruction in the small airways is in itself a predictor of AHR. This composite variable could easily be incorporated into spirometry software in the future.

There were several limitations in this study. Firstly, since this was a retrospective study, the follow-up of patients’ clinical status was not taken into consideration. Since AHR can be present in other respiratory conditions such as chronic obstructive pulmonary disease (COPD), atopic individuals without respiratory symptoms and in smokers, some study participants could have been misdiagnosed [15, 17]. Ideally, follow up of patients’ response to medical treatment would have been desired. Assuming misdiagnosis only makes up a small part of our study sample, it would likely only add some background noise in the analysis and unlikely change the results significantly. The population in this study may be considered young, with an age range of 18–40 years. However, since this study is focused on asthma diagnosis, this age range is relevant. Indeed, many studies performed on adults with asthma show that the average age of asthma diagnosis in adults is at the early 30 s [18].

A major strength in this study is the large sample size and the use of a validation population to challenge the models. However, the method of random split-sampling, that was used for selecting the validation cohort is not the most desirable method for validation [19]. To further verify that the predictive models perform with the similar accuracy independently of sample population, it would have been preferable to use a validation cohort from an entirely different lung function clinic, to further prove the generalizability of the findings.

This study highlights the use of baseline FEF_50%_ %predicted as a negative predictor of AHR, in adults with suspected asthma. By applying a threshold value of ≥ 110% of baseline FEF_50%,_ almost a fifth of all MCTs may potentially be avoided. Prospective studies in the future would give better insight into the use of standard spirometry for predicting AHR and excluding asthma.

## Conclusions

Baseline spirometry parameters may be used as tools for predicting airway hyper-responsiveness. FEF50% proved to be useful as a negative predictor when applying FEF50% ≥ 110% as a cutoff level for exclusion of airway hyper-responsiveness, reducing the requirement for Methacholine Challenge Tests by approximately 20%, and thus FEF50% may be particularly useful to reduce the burden of unnecessary MCTs.

## Supplementary Information

**Additional file 1.** **Figure S1.** Angle β definition.**Table 1s.** Pulmonary lung function characteristics at baseline for primary and test cohort based on GLI equations. **Table 2s.** Comparison of baseline spirometry parameters in patients with positive versus negative methacholine challenge test in the primary cohort based on GLI equations. **Figure S2.** ROC-curves for derivation cohort according to GLI equations. **Figure S3.** ROC-curves for derivation cohort according to GLI equations with z-scores. **Figure S4.** ROC-curve FEV1 for the derivation cohort according to GLI equations. **Figure S5.** ROC-curve FEV1 z-score for the derivation cohort according to GLI equations. **Table 3s.** Diagnostic performance of baseline FEV1 % predicted for different cut-off values, obtained by ROC analysis to predict methacholine responsiveness (PC20<16 mg/ml). **Figure S6.** ROC-curves for validation cohort. **Figure S7.** ROC curve of combined variable model for validation cohort.

## Data Availability

The datasets used and/or analysed during the current study are available from the corresponding author on reasonable request.
