# Supplementary material for: Baseline spirometry parameters as predictors of airway hyperreactivity in adults with suspected asthma
Source: BMC Pulm Med. 2021 May 6;21:153. doi: 10.1186/s12890-021-01506-6 (PMC8101108; doi:10.1186/s12890-021-01506-6)
Supplement: Supplementary file 1 — Additional file 1. Figure S1. Angle β definition.Table 1s. Pulmonary lung function characteristics at baseline for primary and test cohort based on GLI equations. Table 2s. Comparison of baseline spirometry parameters in patients with positive versus negative methacholine challenge test in the primary cohort based on GLI equations. Figure S2. ROC-curves for derivation cohort according to GLI equations. Figure S3. ROC-curves for derivation cohort according to GLI equations with z-scores. Figure S4. ROC-curve FEV1 for the derivation cohort according to GLI equations. Figure S5. ROC-curve FEV1 z-score for the derivation cohort according to GLI equations. Table 3s. Diagnostic performance of baseline FEV1 % predicted for different cut-off values, obtained by ROC analysis to predict methacholine responsiveness (PC20<16 mg/ml). Figure S6. ROC-curves for validation cohort. Figure S7. ROC curve of combined variable model for validation cohort. [file 12890_2021_1506_MOESM1_ESM.doc]

**SUPPLEMENTARY INFORMATION**

**TITLE: Baseline Spirometry Parameters as Predictors of Airway Hyperreactivity in Adults with Suspected Asthma**

**Authors’ full names:** Michael Peled^1,2*^, David Ovadya^1,3*^, Jennifer Cohn^4*^, Lior Seluk^1^, Teet Pullerits^5^, Michael J. Segel^1,2*^, Amir Onn^1*^

**Authors’ affiliation(s):**

^1^Institute of Pulmonary Medicine, Chaim Sheba Medical center, Israel; ^2^Sackler Faculty of Medicine, Tel-Aviv University, Tel-Aviv, Israel; ^3^Department of Respiratory Care and Rehabilitation, Chaim Sheba Medical Center, Israel; ^4^Sahlgrenska Academy, Faculty of Medicine, University of Gothenburg, Sweden; ^5^Department of Asthma and Allergology, Sahlgrenska University Hospital, Sweden.

* These authors contributed equally to this study.

**Figure S1***-* **Angle β definition**

**
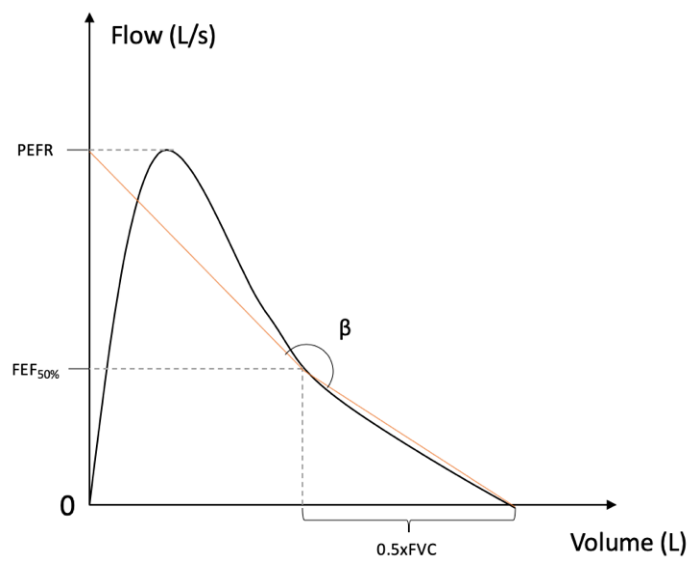
**

Supplemental Figure 1. Angle β defined on a maximal expiratory flow volume curve. PEFR, peak expiratory flow rate; FVC, forced vital capacity; FEF_50%_, forced expiratory flow at 50% of FVC.

| **Table 1s.**  **P**ulmonary lung function characteristics at baseline for primary and test cohort **based on GLI equations**. | | | | | | | | |
| --- | --- | --- | --- | --- | --- | --- | --- | --- |
|  | Primary Cohort (n=2237) | |  | Test Cohort (n=746) | |  | |  |
|  | Mean | SD | Mean | | SD | | *P*-value | |
| FEV_1_/FVC (%pred) GLI | 0.98 | 0.09 | 0.98 | | 0.08 | | 0.72 | |
| FEV_1_ (%pred) GLI | 0.90 | 0.10 | 0.89 | | 0.10 | | 0.55 | |
| FEF_50%_ (%pred) GLI | - | - | - | | - | | - | |
| FVC (%pred) GLI | 0.91 | 0.10 | 0.91 | | 0.11 | | 0.76 | |
| FEF_25-75%_ (%pred) GLI | 0.83 | 0.28 | 0.83 | | 0.27 | | 0.80 | |
| z-score FEV_1_/FVC | -0.15 | 1.14 | -0.17 | | 1.08 | | 0.58 | |
| z-score FEV_1_ | -0.87 | 0.88 | -0.89 | | 0.87 | | 0.53 | |
| z-score FVC | -0.76 | 0.87 | -0.77 | | 0.89 | | 0.74 | |
| z-score FEF_25-75%_ | -0.81 | 1.28 | -0.82 | | 1.23 | | 0.88 | |
|  | | | | | | | | |

| **Table 2s.** Comparison of baseline spirometry parameters in patients with positive versus negative methacholine challenge test in the primary cohort **based on GLI equations**. |
| --- |

|  | Methacholine Negative (n=1932) | | |  | | Methacholine Positive (n=305) | | |  | |  |
| --- | --- | --- | --- | --- | --- | --- | --- | --- | --- | --- | --- |
|  | Mean | | SD | | Mean | | SD | *P*-value | | | |
| FEV_1_/FVC ratio (%pred) GLI | | 0.99 | 0.09 | | 0.94 | | 0.09 | | | < 0.01 | |
| FEV_1_ (%pred) GLI | | 0.91 | 0.10 | | 0.85 | | 0.11 | | | < 0.01 | |
| FVC (%pred) GLI | | 0.91 | 0.10 | | 0.90 | | 0.11 | | | 0.08 | |
| FEF_25-75%_ (%pred) GLI | | 0.84 | 0.28 | | 0.82 | | 0.27 | | | 0.17 | |
| z-score FEV_1_/FVC | | -0.07 | 1.13 | | -0.67 | | 1.06 | | | < 0.01 | |
| z-score FEV_1_ | | -0.80 | 0.85 | | -1.26 | | 0.93 | | | < 0.01 | |
| z-score FVC | | -0.75 | 0.86 | | -0.85 | | 0.95 | | | 0.08 | |
| z-score FEF_25-75%_ | | -0.80 | 1.28 | | -0.90 | | 1.26 | | | 0.21 | |
| Methacholine positive defined as PC20≤16 mg/ml; PC20, Concentration of methacholine causing a 20% decrease in FEV_1_; FEV_1_, forced expiratory volume in 1 s; FVC, forced vital capacity; FEF_25–75%_, forced expiratory flow at 25–75% of FVC; Relative values of spirometry parameters are given as percentage of the predicted value (% pred). | | | | | | | | | | | |

**Figure S2 - ROC-curves for derivation cohort according to GLI equations**


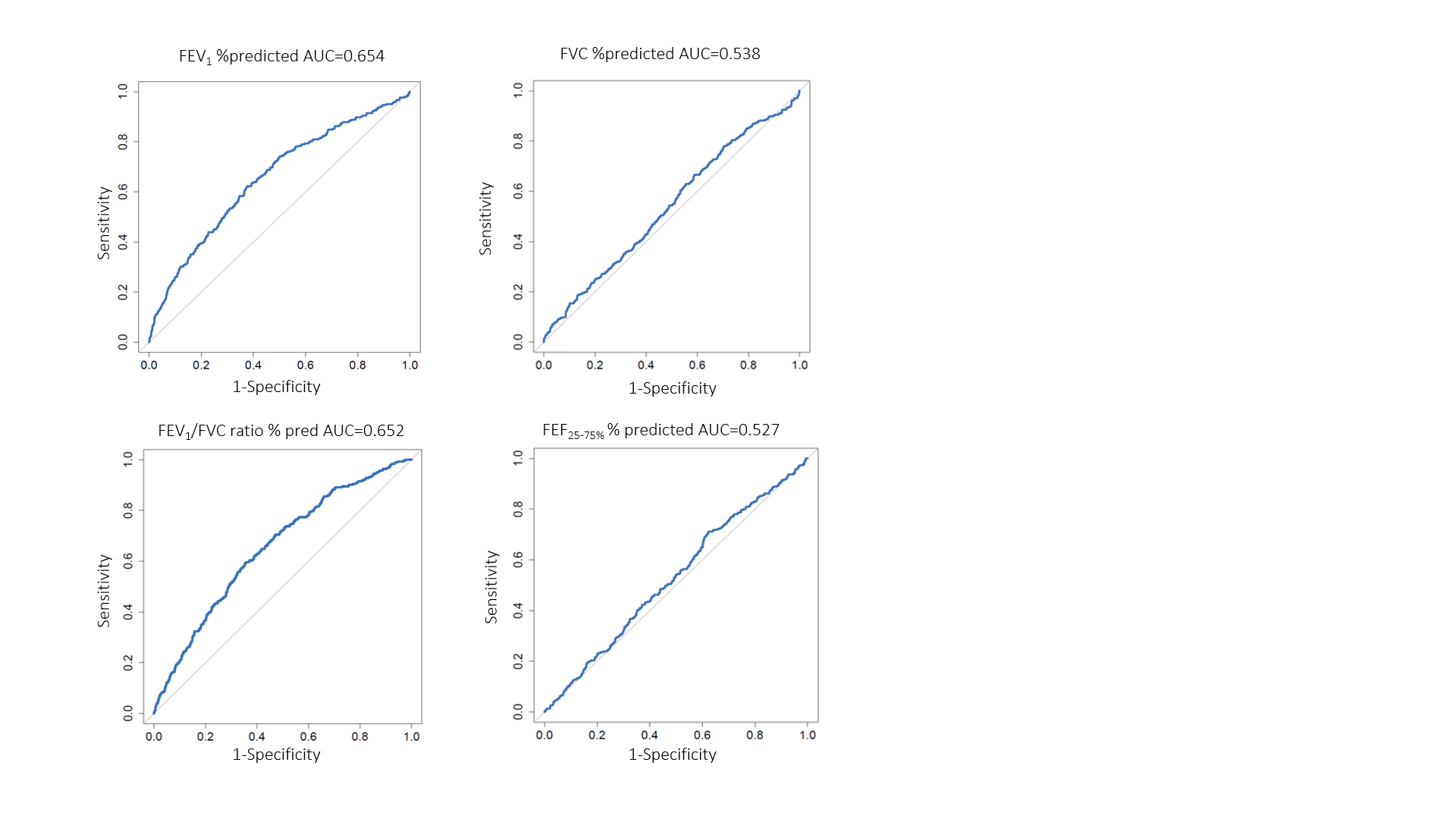


a

b

c

d

Supplemental Figure 2. Receiver operating characteristics (ROC) curve for; a) baseline forced expiratory volume in 1 s, FEV1 %predicted; b) forced vital capacity, FVC %predicted; c) FEV1/FVC; d) forced expiratory flow at 25–75% of FVC, FEF_25-75%_ %predicted in the derivation cohort as predictors of methacholine responsiveness using the GLI equations. AUC=Area under the curve. Line of unity is represented by a grey line, corresponding to AUC=0.5.

**Figure S3 - ROC-curves for derivation cohort according to GLI equations with z-scores**

a

b


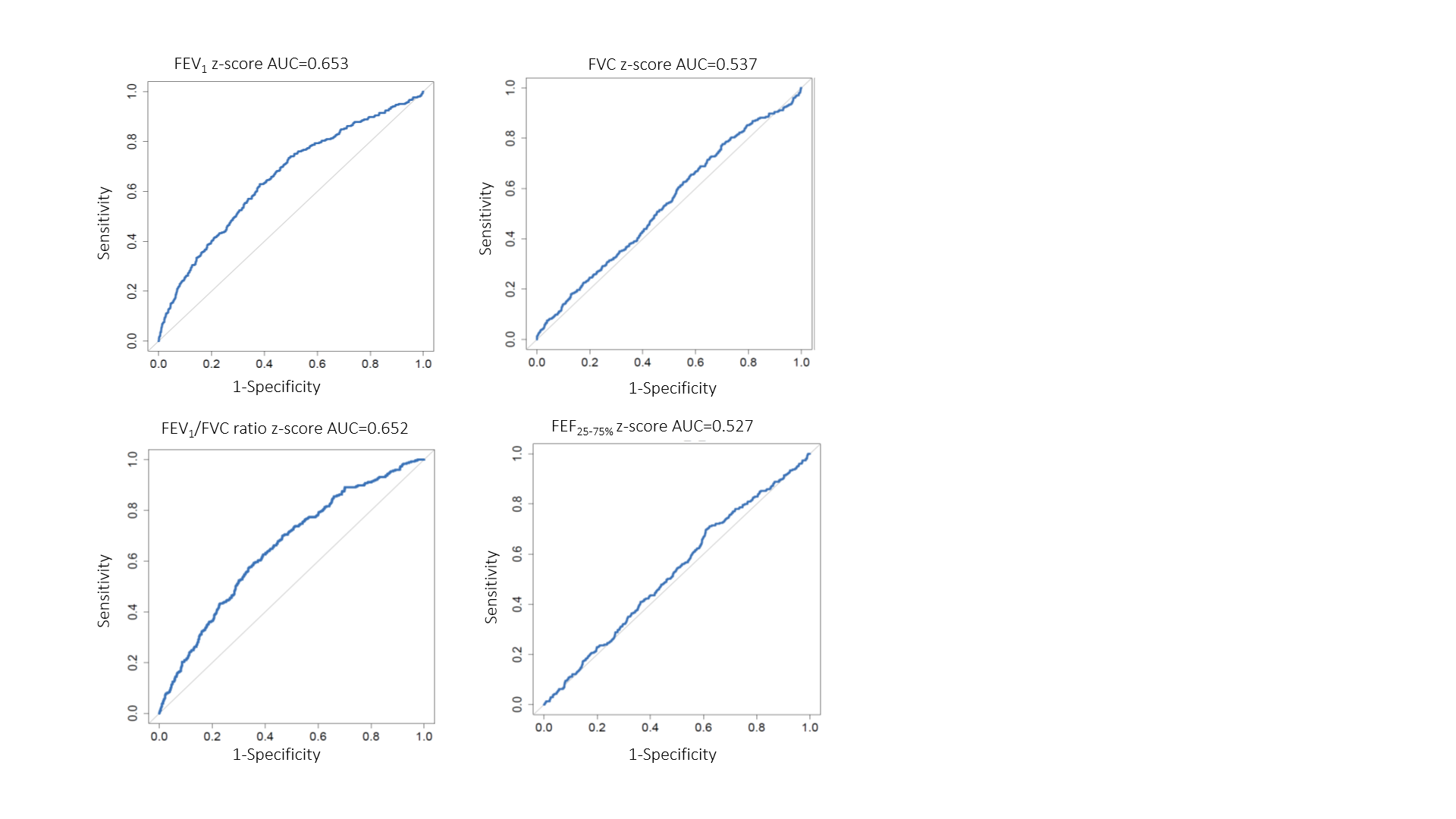


c

d

Supplemental Figure 3. Receiver operating characteristics (ROC) curve for; a) baseline forced expiratory volume in 1 s, FEV1 z-score; b) forced vital capacity, FVC z-score; c) FEV1/FVC; d) forced expiratory flow at 25–75% of FVC, FEF_25-75%_ z-score in the derivation cohort as predictors of methacholine responsiveness using the GLI equations. AUC=Area under the curve. Line of unity is represented by a grey line, corresponding to AUC=0.5.

**Figure S4 - ROC-curve FEV1 for the derivation cohort according to GLI equations**


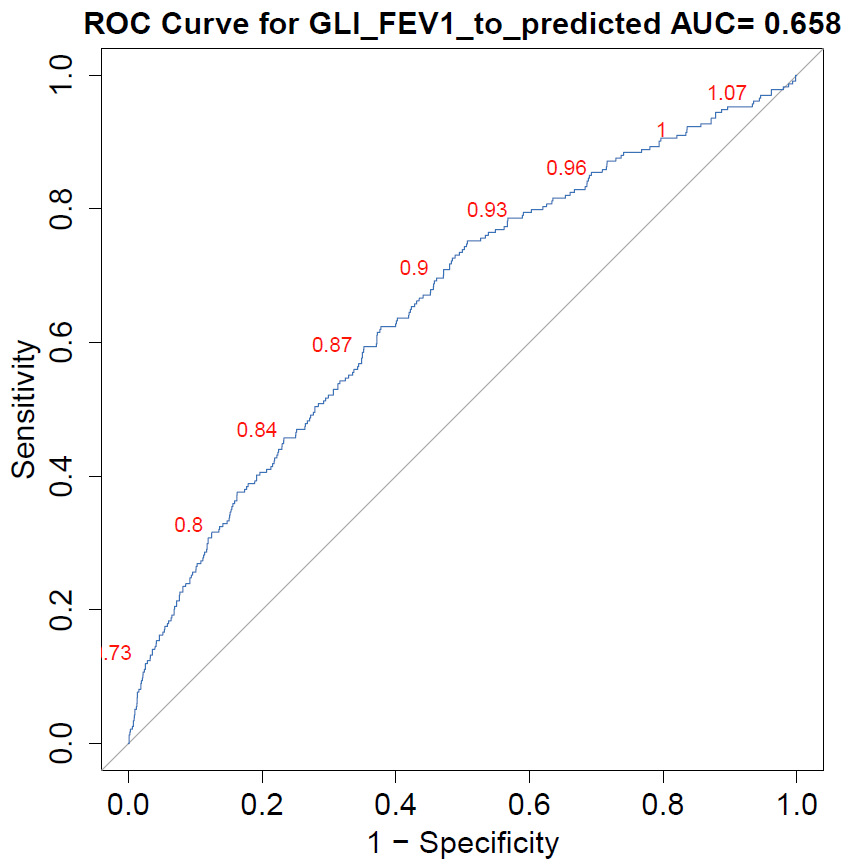


Supplemental Figure 4. Receiver Operating Characteristics (ROC) curve for forced expiratory volume at 1 second of FVC, FEV1%predicted, in the derivation cohort as a predictor of methacholine responsiveness. Values in red represent the respective FEV1 %predicted cut-off values. AUC=Area under the curve. Line of unity is represented by a grey line, corresponding to AUC=0.5.

**Figure S5 - ROC-curve FEV1 z-score for the derivation cohort according to GLI equations**

Supplemental Figure 5. Receiver Operating Characteristics (ROC) curve for forced expiratory volume at 1 second of FVC, FEV1 z-score, in the derivation cohort as a predictor of methacholine responsiveness. Values in red represent the respective FEV1 z-score cut-off values. AUC=Area under the curve. Line of unity is represented by a grey line, corresponding to AUC=0.5.

| Table 3**s**. Diagnostic performance of baseline FE**V1** % predicted for different cut-off values, obtained by ROC analysis to predict methacholine responsiveness (PC20<16 mg/ml). | | | | | |
| --- | --- | --- | --- | --- | --- |
| Cut-off | Sensitivity (%) | Specificity (%) | PPV (%) | NPV (%) |  |
| GLI_FEV1%Predicted: |  |  |  |  |  |
| <120% | 99.10 | 0.10 | 13.60 | 60 |  |
| <115% | 98.70 | 0.80 | 13.50 | 73.70 |  |
| <110% | 97.90 | 2.80 | 13.70 | 88.50 |  |
| GLI_FEV1 z-score: |  |  |  |  |  |
| <1.5 | 99 | 1 | 13.60 | 76.90 |  |
| <1.0 | 98 | 2 | 13.60 | 85.40 |  |
| <0.5 | 96 | 6 | 13.90 | 90.20 |  |
| Sensitivity, specificity, positive predictive value (PPV), negative predicted value (NPV). | | | | | |


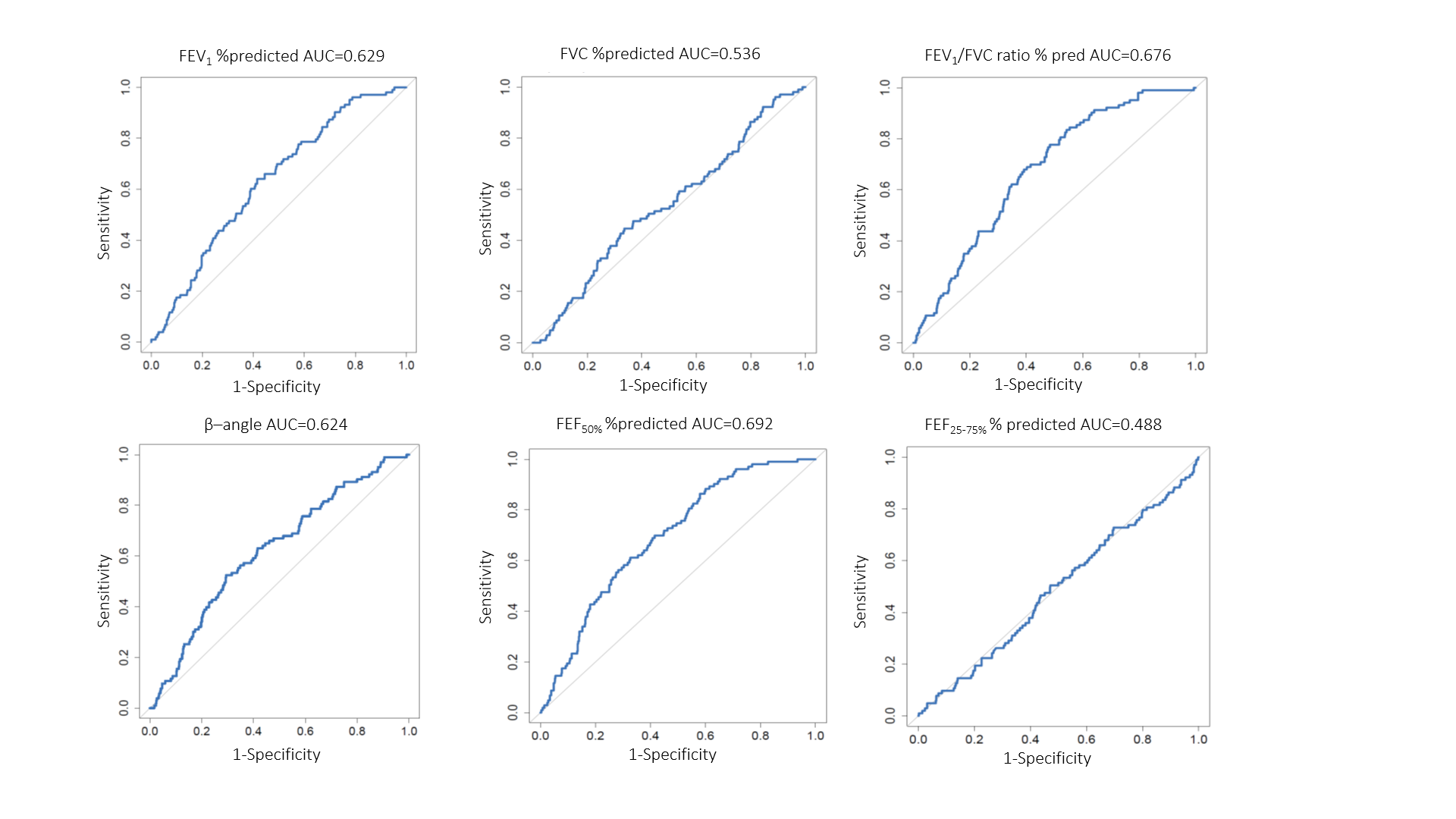
**Figure S6 - ROC-curves for validation cohort**

a

b

c

d

f

e

Supplemental Figure 6. **ROC-curves for validation cohort.** Replication of findings: Receiver operating characteristics (ROC) curve for; a) baseline forced expiratory volume in 1 s, FEV_1_ %predicted; b) forced vital capacity, FVC %predicted; c) FEV_1_/FVC; d) angle β; e) forced expiratory flow at 50% of FVC, FEF_50%_ %predicted; f) forced expiratory flow at 25–75% of FVC, FEF_25-75%_ %predicted in the validation cohort as predictors of methacholine responsiveness. AUC=Area under the curve. Line of unity is represented by a grey line, corresponding to AUC=0.5.


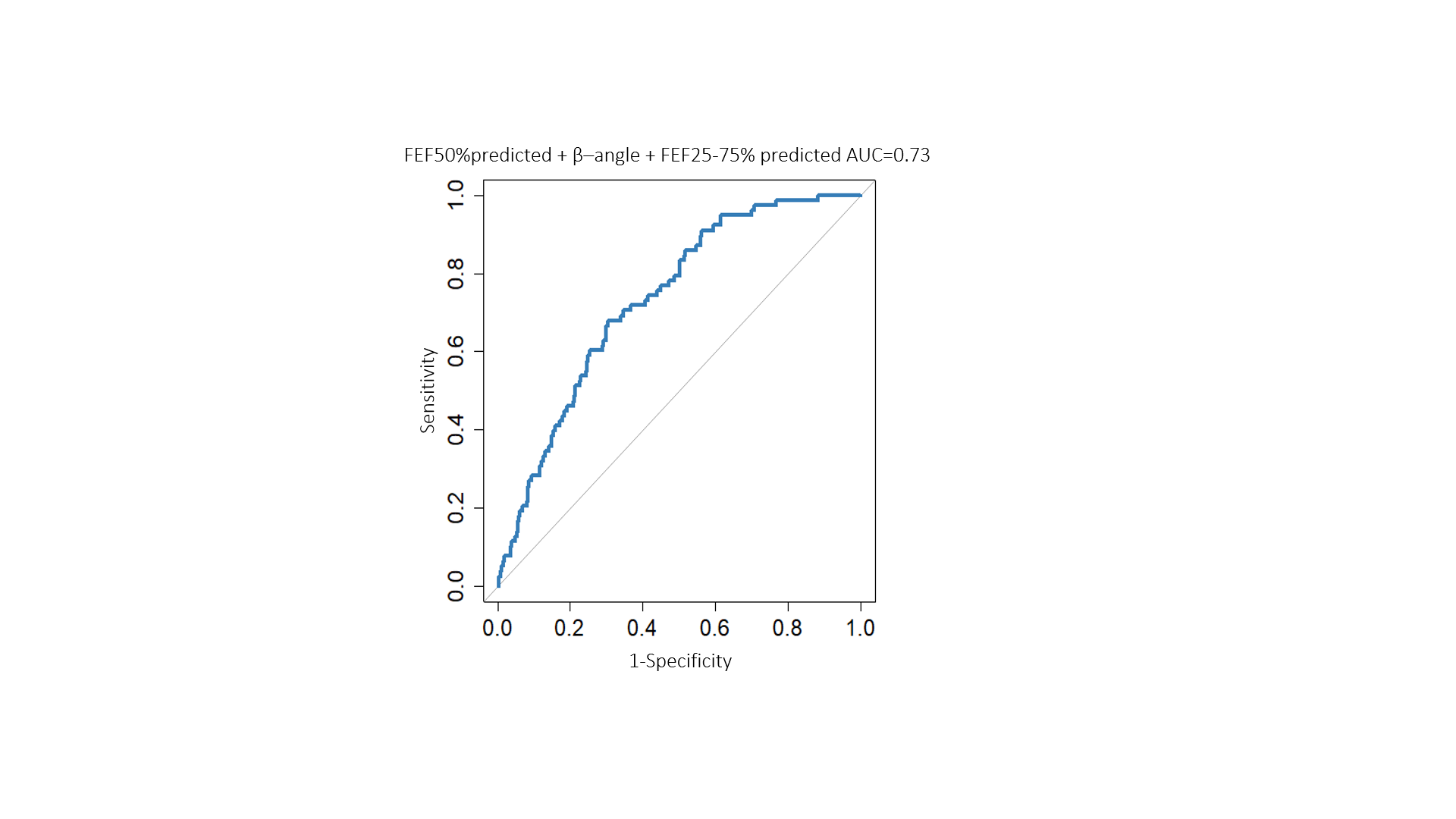
**Figure S7 - ROC** **curve of combined variable model for validation cohort.**

Supplemental Figure 7. **ROC curve of combined variable model for validation cohort.** Receiver operating characteristics (ROC) curve for model combining forced expiratory flow at 50% of FVC, FEF_50%_ %predicted, angle β and forced expiratory flow at 25-75% of FVC, FEF_25-75%_ %predicted as a predictor of methacholine responsiveness in the validation cohort. AUC=Area under the curve. Line of unity is represented by a grey line, corresponding to AUC=0.5.
